# Spleen tyrosine kinase (SYK) inhibition suppresses growth of gastrointestinal neuroendocrine tumor cells: a pilot study in two cell lines

**DOI:** 10.1038/s41417-025-00979-5

**Published:** 2025-10-16

**Authors:** Angeliki Ditsiou, Lara Toffoli, Viviana Vella, Francesca D’Este, Teresa Gagliano

**Affiliations:** 1 Cancer Cell Signalling Lab, Udine, Italy; 2 Department of Medicine, University of Udine, Udine, Italy; 3 School of Life Science, University of Sussex, Brighton, UK

**Keywords:** Cell biology, Gastric cancer

## Abstract

Gastrointestinal neuroendocrine tumors (GI-NETs) lack effective targeted options beyond somatostatin analogs and mTOR inhibitors. Spleen tyrosine kinase (SYK) is a non-receptor kinase with emerging roles in solid tumors and available small-molecule inhibitors. We explored whether SYK is a plausible therapeutic target in GI-NET using two human cell lines. SYK expression in GI-NET cells was confirmed by immunofluorescence. Cells were exposed to a selective SYK inhibitor (BI-1002494), and proliferation was quantified using both 2D and 3D models. Both GI-NET models expressed SYK and exhibited reduced growth upon SYK blockade, with dose-dependent suppression of viability and increased cytotoxicity relative to vehicle. In spheroid assays, morphologic changes and reduced size were observed. These pilot data suggest SYK as a targetable vulnerability in GI-NET and support formal dose–response studies, genetic validation, and combination strategies with standard-of-care agents. Given the clinical availability of SYK inhibitors, these findings provide a rationale for translational studies in GI-NET.

## Introduction

Gastrointestinal neuroendocrine tumors (GI-NETs) are rare and biologically heterogeneous, with a consistently increasing incidence. Despite this trend, therapies remain limited because resistance is common, and diagnosis often occurs at advanced disease stages [1–3].

Spleen tyrosine kinase (SYK) integrates immunoreceptor and integrin signaling. Although best studied in hematologic malignancies, in solid tumor SYK has been reported to play a major role in tumor cell survival and proliferation, underscoring its tumor-promoting function and therapeutic potential, including in endocrine related cancer, suggesting context-dependent oncogenic functions [4].

Currently the role of SYK in GI-NET biology has not been investigated. Our aim is to test whether inhibition of SYK suppresses growth of GI-NET cells, providing pilot evidence for SYK as a therapeutic target.

## Brief descriptions of materials and methods

### Cell lines and culture

Human GI-NET cell line GOT1, gift of Ola Nilsson Group, [5] and a colorectal adenocarcinoma cell line with neuroendocrine features COLO320DM were maintained under standard conditions as previously described. Cells were kept at low passage, routinely authenticated by morphology/growth characteristics, and regularly tested for mycoplasma [6].

### Compound

The SYK inhibitor BI-1002494 was obtained from Boehringer Ingelheim via opnMe (https://www.opnme.com).

### Antibodies

Rabbit anti-human SYK (ABclonal) and ABflo® 647–conjugated mouse anti-rabbit IgG (ABclonal) were used for immunofluorescence.

### 2D/3D viability

For 2D assays, 3 × 10³ cells/well were seeded in 96-well plates (low-serum medium), treated for 72 h, fixed in 4% paraformaldehyde, stained with 0.1% crystal violet, air-dried overnight, solubilized in 10% acetic acid, and read spectrophotometrically. For 3D assays, viability was quantified using CellTiter-Glo® 3D (Promega) according to the manufacturer’s protocol, with ATP-dependent luminescence measured on a plate reader [7].

### Colony formation

COLO320DM cells (3 × 10³/well, 6-well plates; low-serum) were treated for 14 days with medium refreshed every 3–4 days, stained with crystal violet, and colonies quantified in ImageJ [8].

### Caspase-3/7 activity

Cells were treated for 24 h and analysed using Caspase-Glo® 3/7 (Promega) per the manufacturer’s instructions; luminescence was recorded on a plate reader.

### Invasion

Matrigel-coated transwells (8 µm pores) received 5 × 10⁵ cells/insert (low-serum) plus treatment; 20% FBS in the lower chamber served as chemoattractant. After 24 h, inserts were fixed (4% paraformaldehyde), stained with crystal-violet, and invading cells on the lower membrane surface were counted microscopically [9].

### Immunofluorescence

Cells on coverslips were fixed (4% paraformaldehyde), permeabilized (0.1% Triton X-100), blocked (5% BSA), incubated with anti-SYK (overnight), then secondary antibody and Hoechst. Slides were mounted with anti-fade medium and imaged by confocal microscopy [10].

### RNA and qPCR

RNA was isolated (PureLink™), reverse-transcribed (High-Capacity kit), and SYBR-based qPCR run on QuantStudio™ 1; β-ACTIN served as control. Primer sequences:

*BACT-FW: CGCCGCCAGCTCACCATG*

*BACT-REV CACGATGGAGGGGAAGACGG*

*E-CADH-FW GGCAGCTAATACAGACTATAAG*

*E-CADH-REV TGTGAAGTTTGGATTGACAG*

### Statistics

Analyses were performed in GraphPad Prism 10; tests are specified in figure legends, with *p* < 0.05 considered significant. Unless noted, experiments used ≥3 biological replicates (qPCR: ≥2 biological, 3 technical replicates).

## Results

### SYK inhibition reduces cell viability and increases apoptosis in GI-NET cell lines

**Fig. 1:**
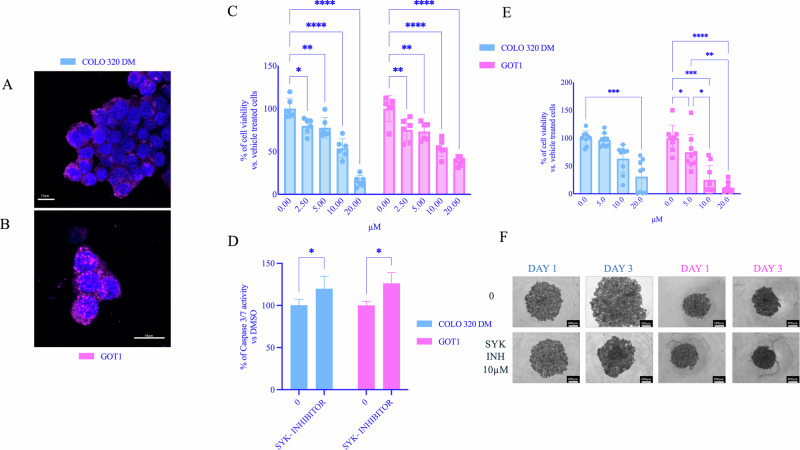
Effects of SYK inhibition on GI-NETs cells 2D and 3D culture. SYK expression was detected in both COLO320DM (**A**) and in GOT1 (**B**) cells by immunofluorescence staining (SYK is stained magenta, while nuclei are shown in blue). Effects of SYK inhibition on 2D and 3D NETs cell culture. **C**, **E** COLO320DM and GOT1 cells were seeded in 96 well plates and incubated for 72 h with increasing concentration of SYK inhibitor, control cells were treated with a vehicle (DMSO). Cell viability in 2D assays (**C**) was assessed by crystal violet in three independent experiments with at least six replicates each, and it is expressed as the mean ± SEM. *P < 0.05 vs vehicle cells; ***P < 0.0001 vs vs vehicle cells. ****P < 0.00001 vs vs vehicle cells. Cell viability of 3D cells (**E**) was measured as luminescent output in three independent experiments with at least two replicates each, and it is expressed as the mean ± SEM **P < 0.01 vs vs vehicle cells, ****P < 0.00001 vs vs vehicle cells, significance was calculated by two-way ANOVA using Dunnett’s multiple comparison test. **F** Representative images of 3D spheroids at baseline, Day 1, and Day 3 of treatment. **D** Caspase activity was measured as luminescent output in two independent experiments with two replicates each, and it is expressed as the mean ± SEM. ***P < 0.01 vs vs vehicle cells, ****P < 0.00001 vs vehicle cells, significance was calculated by two-way ANOVA using Dunnett’s multiple comparison test.

SYK expression was analysed by immunofluorescence, revealing a marked protein expression in both cell lines (Fig. 1A, B). To evaluate the impact of SYK inhibition on the viability of GI-NET cells, COLO320DM and GOT1 cells were treated with increasing concentrations of SYK inhibitor (0–10/20 µM range). In 2D cultures, a significant dose-dependent reduction in cell viability was observed in both COLO320DM and GOT1 cells, as assessed by the crystal violet assay (Fig. 1C). A 2.5 µM concentration of the compound was sufficient to significantly reduce cell viability. In 3D spheroid cultures of COLO320DM and GOT1, treatment with SYK inhibitor did not decrease its efficacy in reducing cell viability, with statistical significance requiring concentrations of 5–10 µM (Fig. 1E). In addition, the spheres of GI-NETs cell lines showed a reduction in volume upon treatment with SYK inhibitor (Fig. 1F).

Increased caspase activity confirmed apoptosis induction following treatment with SYK inhibitor (Fig. 1D). These results suggest that SYK inhibition activates apoptotic pathways contributing to the observed reduction in cell viability.

### SYK inhibition decreases invasion, clonogenicity and increases E-cadherin expression in GI-NET cells

**Fig. 2:**
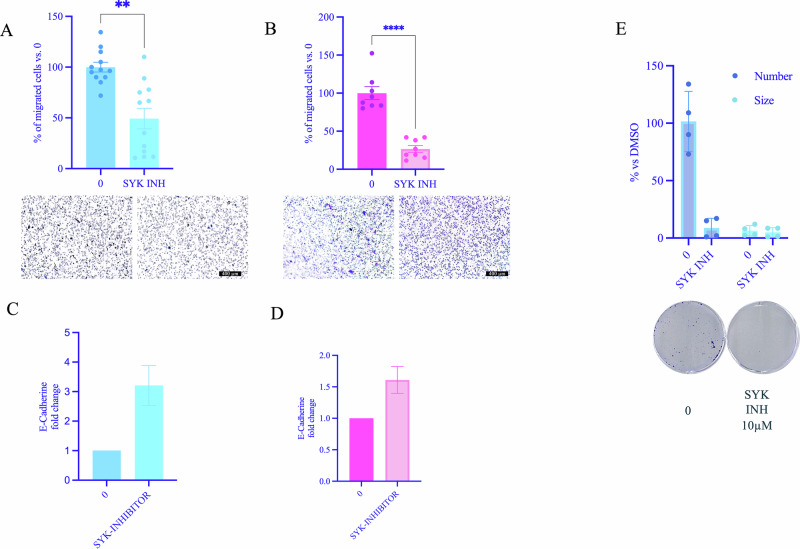
SYK inhibitor decreases clonogenic and invasive potential of GI-NETs by increasing E-Cadherin levels. For the invasion assay of COLO320DM (**A**) and GOT1 (**B**), cells were seeded on the Matrigel-coated upper chamber of the transwell. Twenty-four hours later, migrated cells were fixed, stained, and counted (*n* = 3 independent experiments, minimum 2 technical replicates). qRT-PCR of E-cadherin expression levels in COLO320DM (**C**) and GOT1 (**D**) cells following treatment with SYK inhibitor. Significance was calculated using one-way ANOVA. Data are expressed as mean ± SEM; **P < 0.01, ****P < 0.0001 vs. DMSO, significance was calculated by Anova using Dunnett’s multiple comparison test. Changes in colony number and dimension of COLO320DM (**E**) after treatment with SYK inhibitor, with representative images of colony formation assays. Colonies were quantified using Image J Software and results show the percentage of colonies formed after treatment with the indicated concentrations of the drug (surviving fraction), corrected according to the plating efficiencies of the corresponding controls. Data are shown as mean ± SEM of at least three independent experiments, *P < 0.05 vs vehicle cells significance was calculated by two-way ANOVA using Šidák’s multiple comparison test.

Invasion assays demonstrated that treatment with SYK inhibitor markedly reduced the invasive ability of COLO320DM (Fig. 2A) and GOT1 (Fig. 2B) cells compared to DMSO-treated controls, indicating that SYK activity is involved in maintaining invasive properties of GI-NET cells. Consistently, increased level of E-cadherin was detected in both cell lines upon treatment with SYK inhibitor (Fig. 2C, D). We next examined the effect of SYK inhibition on colony-forming potential. Treatment with SYK inhibitor reduced the colony-forming ability of COLO320DM cells (Fig. 2E), suggesting a role for SYK in sustaining their growth potential.

## Discussion and future perspectives

These data provide preliminary evidence that SYK supports growth and invasiveness in GI‑NET models. Pharmacologic SYK blockade reduces viability, invasion, and clonogenicity while increasing E‑cadherin expression, consistent with a less aggressive phenotype.

In solid tumors, SYK connects receptor and integrin cues to PI3K/AKT, NF-κB, and context-dependent STAT signaling that governs survival and invasion. This supports SYK inhibition as the basis of our observed effects and justify focused pathway mapping in GI-NETs [11]. Given the availability of clinically tested SYK inhibitors, key next steps include confirming target engagement (e.g., p‑SYK inhibition), genetic validation of SYK dependence (e.g. by siRNA/CRISPRi experiments), and testing combinations with somatostatin analogs or mTOR inhibitors in patient-derived models and in vivo to inform biomarker‑guided early‑phase trials.
